# *polo* Is Identified as a Suppressor of *bubR1* Nondisjunction in a Deficiency Screen of the Third Chromosome in *Drosophila melanogaster*

**DOI:** 10.1534/g3.111.000265

**Published:** 2011-07-01

**Authors:** Sofia Sousa-Guimarães, Claudio Sunkel, Nicolas Malmanche

**Affiliations:** * Instituto de Biolgia Molecular e Celular, Universidade do Porto, Porto, Portugal; † Instituto de Ciências Biomédicas de Abel Salazer, Univeriadde do Porto, Porto, Portugal

## Abstract

We have previously characterized an EMS-induced allele of the *bubR1* gene (*bubR1^D1326N^*) that separates the two functions of BubR1, causing meiotic nondisjunction but retaining spindle assembly checkpoint activity during somatic cell division in *Drosophila melanogaster*. Using this allele, we demonstrate that *bubR1* meiotic nondisjunction is dosage sensitive, occurs for both exchange and nonexchange homologous chromosomes, and is associated with decreased maintenance of sister chromatid cohesion and of the synaptonemal complex during prophase I progression. We took advantage of these features to perform a genetic screen designed to identify third chromosome deficiencies having a dominant effect on *bubR1^D1326N^*/*bubR1^rev1^* meiotic phenotypes. We tested 65 deficiencies covering 60% of the third chromosome euchromatin. Among them, we characterized 24 deficiencies having a dominant effect on *bubR1^D1326N^*/*bubR1^rev1^* meiotic phenotypes that we classified in two groups: (1) suppressor of nondisjunction and (2) enhancer of nondisjunction. Among these 24 deficiencies, our results show that deficiencies uncovering the *polo* locus act as suppressor of *bubR1* nondisjunction by delaying meiotic prophase I progression and restoring chiasmata formation as observed by the loading of the condensin subunit SMC2. Furthermore, we identified two deficiencies inducing a lethal phenotype during embryonic development and thus affecting BubR1 kinase activity in somatic cells and one deficiency causing female sterility. Overall, our genetic screening strategy proved to be highly sensitive for the identification of modifiers of BubR1 kinase activity in both meiosis and mitosis.

Mitosis is a process that results in the production of two identical daughter cells from a single cell. At metaphase-anaphase transition, the accuracy of chromosome segregation is ensured by the spindle assembly checkpoint (SAC) that monitors microtubule-kinetochore attachment and prevents mitotic exit until all chromosomes are attached to the bipolar spindle and under tension. The Mad (mitotic arrest deficient) and Bub (budding uninhibited by benomyl) SAC components were first identified in budding yeast through genetic screens designed to isolate mutations which override the mitotic arrest in the presence of microtubule depolymerizing drugs (Hoyt *et al.* 1991; Li and Murray 1991). Immunolocalization studies have shown that these conserved proteins localize to kinetochores that are unattached or under reduced tension (Chen *et al.* 1998; Taylor *et al.* 1998; Logarinho *et al.* 2004). The SAC proteins impose a mitotic arrest by inhibiting the activity of the anaphase-promoting complex/cyclosome (APC/C) that is essential for sister chromatid separation and mitotic exit (Li and Benezra 1996; Taylor and Mckeon 1997; Bernard *et al.* 1998; Gorbsky *et al.* 1998; Basu *et al.* 1999). However, the Bub1-related kinase (BubR1), which displays N-terminal homology with the yeast Mad3 protein and C-terminal homology with the Bub1 kinase domain, is found only in higher eukaryotes (Taylor *et al.* 1998). In addition to its involvement in the SAC, BubR1 is also required for proper mitotic timing, capture, and stabilization of kinetochore-microtubule attachment at prometaphase-metaphase transition (Basu *et al.* 1999; Ditchfield *et al.* 2003; Logarinho *et al.* 2004; Harris *et al.* 2005; Lampson and Kapoor 2005) and for mitotic arrest in the presence of DNA damage (Fang *et al.* 2006). Furthermore, BubR1 is essential to prevent early aging and infertility in mice (Baker *et al.* 2006; Hartman *et al.* 2007; Matsumoto *et al.* 2007).

In contrast to mitosis, meiosis results in the production of haploid gametes from a diploid parental cell. Meiosis involves one single round of DNA replication followed by two sequential rounds of cell division (meiosis I and meiosis II). During meiotic prophase I, homologous chromosomes undergo a complex series of modifications through pairing, synapsis, and exchange to ensure chromosome reduction and sister chromatid separation during meiosis. Nondisjunction (NDJ), the failure to properly segregate the genome during meiosis, produces haploid cells that have unbalanced genetic composition. The vast majority of meiotic segregation errors occur during meiosis in females, and the error rate increases with advancing maternal age. The frequency of missegregation in human oocytes is remarkably high (about 10% of meiosis), and this is thought to be one reason for the high rate of miscarriages (spontaneous abortions) in early stages of pregnancy (Alberts *et al.* 2002).

In contrast to mitosis, where BubR1 has an important role in controlling the metaphase-anaphase transition, it was shown that MAD3/BubR1 has an essential and conserved function during prophase I progression in meiosis. MAD3/BubR1 is required to delay prophase I in response to nonexchange chromosomes in budding yeast (Cheslock *et al.* 2005). In *D. melanogaster*, BubR1 was shown to play an essential role during meiotic progression in both sexes and to prevent missegregation of both chiasmate and achiasmate homologous chromosomes. During oogenesis, BubR1 is also required to maintain the synaptonemal complex (SC) (Malmanche *et al.* 2007). In mice, BubR1-depleted oocytes have a reduced capacity to trigger essential meiotic arrest through destabilization of the APC/C inhibitor Cdh1 (Homer *et al.* 2009). It was also shown that overexpression of exogenous BubR1 arrests oocyte maturation during meiosis I, while dominant-negative BubR1 expression accelerates meiotic progression (Wei *et al.* 2010). These studies indicate an essential requirement for BubR1 function in timing the complex events taking place during meiotic prophase I progression.

In *D. melanogaster*, we have previously characterized a *bubR1* EMS allele with a point mutation in a conserved amino acid essential for its kinase activity, *bubR1^D1326N^* (Malmanche *et al.* 2007). Using this allele, we showed that individuals with *bubR1^D1326N^* in *trans* with two previously characterized alleles, *bubR1^1^* (Basu *et al.* 1999) and *bubR1^rev1^* (Perez-Mongiovi *et al.* 2005), and with *Df(2R)nap9*, a deficiency uncovering *bubR1*, lack any of the visible phenotypes that are characteristic of a defect in mitosis and have a functional SAC. However, during meiosis, *bubR1^D1326N^ trans*-heterozygotes show meiotic NDJ for the autosome and sex chromosomes, with the frequency being BubR1 dosage-dependent. We took advantage of these sensitized genetic backgrounds (Malmanche *et al.* 2007) to perform a genetic screening to identify third chromosome deficiencies displaying suppression or enhancement of X NDJ frequency. For this, we used the third chromosome *D. melanogaster* deficiency kit from the Bloomington *Drosophila* Stock Center and we tested 60% of the third chromosome euchromatin. Our results identified 18 deficiencies that suppress and 6 deficiencies that enhance X NDJ. Among the candidate genes tested, we identified Polo kinase as a strong suppressor of the NDJ phenotype and demonstrate that this effect is related to the maintenance of SC during prophase I progression. We identified one deficiency that causes female sterility and two deficiencies affecting BubR1 kinase activity during embryonic development and somatic cell cycle progression, indicating that other pathways can complement BubR1 kinase activity in an otherwise wild-type genetic background.

## Materials and Methods

### *Drosophila melanogaster* stocks

The third chromosome deficiency kit, as well as some of the mutant alleles, were obtained from the Bloomington *Drosophila* Stock Center.

### Genetic screen

From the 96 deficiency stocks of the third chromosome, we were able to generate and maintain 65 deficiencies in the *bubR1^D1326N^* genetic background. To test their effect on female X NDJ, *bubR1^rev1^/bubR1^D1326N^;Df/+* females were crossed to *C(1;Y)1*, *v f B/O* males, and the resulting X NDJ frequency was calculated from the progeny. From this cross, regular female progeny are *Bar* (*X/C(1;Y)1*, *v f B*) and regular male progeny are non-*Bar* (*X/O*), exceptional female progeny are non-*Bar* (*XX/O*) and exceptional male progeny are *vermilion*, *forked*, and *Bar* (*C(1;Y)1*, *v f B/O*). The X NDJ frequency was calculated using the following formula: X NDJ = (2 × males and females exceptional progeny)/Adjusted total progeny, with the Adjusted total progeny = (2 × males and females exceptional progeny) + normal progeny. The exceptional progeny was multiplied by 2 due to the lethality associated with half of the exceptional progeny (triplo-X and nullo-X).

### Immunofluorescence on ovaries

Ovaries were dissected in cold 1× PBS. Fixation was performed for 20 min in 2% EM grade formaldehyde as described in Page and Hawley (2001), followed by 2 hr permeabilization in 1× PBS - 0,5% Triton X-100–10% calf serum. Primary antibodies used were: guinea pig anti-C(3)G (Page and Hawley 2001), rat anti-SMC1 (Malmanche *et al.* 2007), rabbit anti-SMC2 (Savvidou *et al.* 2005), mouse anti-Orb (Lantz *et al.* 1994). The following secondary antibodies were used at 1:1000: anti-rabbit Alexa Fluor 488, anti-mouse Alexa Fluor 568 and anti-guinea pig Alexa Fluor 647. DNA was detected in 1X PBS containing 1 µg/ml of DAPI. Images were obtained using a Leica TCS SP2 AOBS Confocal Microscope (Leica Microsystems, Heidelberg), deconvolved using Huygens Essential (version 3.0.2pl) and processed with Adobe Photoshop 7.0.

### Immunofluorescence on embryos

Wild-type and *bubR1* mutant embryos were collected and aged at 25°C. Immunodetection on embryos was performed as described in Sullivan *et al.* (2000). Primary antibody used was: anti-phospho H3 rabbit polyclonal, used at 1:500 (Upstate Biotechnology). Secondary antibody used was: anti-rabbit Alexa Fluor 488 used at 1:2000 (Molecular Probes). DNA was detected in 1× PBS containing 1 µg/ml of DAPI. For colchicine treatment, embryos were permeabilized in n-heptane, containing 250 µM colchicine in 1× PBS for 30 min before fixation. Images were obtained using a Leica TCS SP2 AOBS Confocal Microscope (Leica Microsystems, Heidelberg), deconvolved using Huygens Essential (version 3.0.2pl) and processed with Adobe Photoshop 7.0.

## Results

### Analysis of X chromosome NDJ in *bubR1^D1326N^/bubR1^rev1^* females

To determine whether a specific third chromosome deficiency has an effect on *bubR1* X NDJ, first we analyzed the overall variations observed in the control genotype, *X/X;bubR1^D1326N^/bubR1^rev1^*. During the screening procedure, we performed 16 rounds of X NDJ experiments, using for each group a control with the *X/X;bubR1^D1326N^/bubR1^rev1^* genotype. Within these 16 controls, the X NDJ frequency ranges from 16.60% to 35.20%, allowing us to calculate an average frequency of 25.82% with a standard deviation of 4.87% (Table 1). These results indicate an inherent variability in the experimental procedure that reflects uncontrollable environmental effects, as well as sampling errors. Thus, in a first approach, we consider that a given third chromosome deficiency has an effect on *bubR1* X NDJ if the frequency is below 20.95% or above 30.69% of the control values, and if the given deficiency shows the same effect when compared to the control for its specific experimental round. For that, we performed a statistical analysis using the multinomial-Poisson hierarchy model (Zeng *et al.* 2010) to take into account variations in sample size and sampling errors. Using these parameters, we identified 24 deficiencies that affect *bubR1* X NDJ with a 95% of confidence interval.

**Table 1:** NDJ of *X/X ; bubR1^D1326N^/bubR1^rev1^* females of the 16 control experiments

Experiment	Normal Progeny	Exceptional Progeny		Total Adjusted Progeny	X NDJ
	X/XY and X/O	XX/O	O/XY		
1	1869	122	64	2241	16.60%
2	2641	280	40	3281	19.51%
3	1452	149	48	1846	21.34%
4	2885	272	153	3735	22.76%
5	1782	233	33	2314	22.99%
6	1286	111	85	1678	23.36%
7	1138	157	28	1508	24.54%
8	972	130	32	1296	25.00%
9	1545	170	107	2099	26.39%
10	737	102	34	1009	26.96%
11	1324	178	72	1824	27.41%
12	282	45	10	392	28.06%
13	631	79	49	887	28.86%
14	1116	210	44	1624	31.28%
15	775	167	22	1153	32.78%
16	681	166	19	1051	35.20%
				Average	25.82% ± 4.87%

### Third chromosome deficiencies modifying *bubR1* X chromosome NDJ

The third chromosome deficiency kit that we originally used included 96 deficiencies. From these, we were able to generate 65 stocks in the appropriate *bubR1^D1326N^* genetic background that covers 60% of the third chromosome euchromatin. As stated above, we performed 16 rounds of experiments to screen these 65 deficiencies (Table 2 and supporting information, Table S1). Using the appropriate parameters for comparison with controls (see above), we identified 6 deficiencies that enhance and 18 deficiencies that suppress the *bubR1* X NDJ. We also identified one deficiency (*Df(3L)ED4674*) causing sterility in *bubR1^D1326N^/bubR1^rev1^* females and two deficiencies (*Df(3L)BSC10* and *Df(3R)BSC56*) inducing a zygotic developmental arrest. Following the first round of screening, we performed a second round of genetic screens using overlapping deficiencies and mutant alleles of candidate genes for three deficiencies that enhance and two that suppress *bubR1* X NDJ, for the deficiency that causes female sterility, and for the two deficiencies that induce an embryonic lethal phenotype. We also performed complementation tests using a number of overlapping deficiencies for the remaining enhancers and suppressors to confirm the breakpoints of the deficiencies in the stocks we established and used to test *bubR1* X NDJ (Table S2).

**Table 2:** Deficiencies that enhance or suppress X NDJ of *X/X;bubR1^D1326N^/bubR1^rev1^* females

	Cytogenetic Breakpoints	Normal ProgenyX/XY and X/O	Exceptional Progeny		Total Adjusted Progeny		Δ With Matched Control*^a^*	Δ With Average Control*^b^*	
Deficiency Name			XX/O	O/XY		X NDJ			Candidate
Enhancer deficiencies									
* Df(3L)BSC23*	62E8;63B5-6	550	127	16	836	34.21%*^c^*	14.70%	8.39%	Mrtf; aly
* Df(3L)BSC13*	66B12-C1;66D2-4	841	281	14	1431	41.23%*^c^*	18.24%	15.41%	mtrm
* Df(3L)Pc-2q*	78C5-6;78E3-79A1	323	109	15	571	43.43%*^c^*	18.43%	17.61%	pzg; Pc; SAK
* Df(3R)ED5177*	83B4;83B6	462	105	16	704	34.38%*^c^*	9.84%	8.56%	asl
* Df(3R)ED5780*	89E11;90C1	61	22	4	113	46.02%*^c^*	17.16%	20.20%	cal1; ald
* Df(3R)D605*	97E2;98A3-4	274	85	15	474	42.19%*^c^*	17.19%	16.37%	Klp98A
Suppressor deficiencies									
* Df(3L)pbl-X1*	65F6;66B7-8	780	30	19	878	11.16%*^c^*	−11.83%	−14.66%	Pdp1;pbl; Arp66B
* Df(3L)ZP1*	66A17-20;66C1-5	968	81	21	1172	17.41%*^c^*	−5.58%	−8.41%	pbl; Arp66B
* Df(3L)BSC8*	74D3-75A1;75B2-5	687	56	17	833	17.53%*^c^*	−7.01%	−8.29%	CycT
* Df(3L)ED4858*	76D3;77C1	566	23	13	638	11.29%*^c^*	−17.58%	−14.53%	polo
* Df(3L)rdgC-co2*	77A1;77D1	686	37	11	782	12.28%*^c^*	−14.68%	−13.54%	polo
* Df(3L)ED4978*	78D5;79A2	1712	69	27	1904	10.08%*^c^*	−6.52%	−15.74%	
* Df(3L)Ten-m-AL29*	79C1-3;79E3-8	1079	64	19	1245	13.33%*^c^*	−6.17%	−12.49%	
* Df(3R)Tpl10*	83C1-2;84B1-2, 83D4-5;84A4-5;98F1-2	647	20	22	731	11.49%*^c^*	−8.02%	−14.33%	bcd
* Df(3R)GB104*	85D12;85E10	189	2	1	195	3.08%*^c^*	−13.52%	−22.74%	βTub85D; hyd; αTub85E; topi
* Df(3R)ED5559*	86E11;87B11	270	0	0	270	0.00%*^c^*	−21.34%	−25.82%	aur; ssp5
* Df(3R)sbd105*	88F9-89A1;89B9-10	1022	106	23	1280	20.16%*^c^*	−15.05%	−5.66%	c(3)G; msps
* Df(3R)ED5942*	91F12;92B3	725	78	18	917	20.94%*^c^*	−6.47%	−4.88%	
* Df(3R)BSC55*	94D2-10;94E1-6	613	43	20	739	17.05%*^c^*	−10.36%	−8.77%	sav
* Df(3R)mbc-30*	95A5-7;95C10-11	181	8	1	199	9.05%*^c^*	−18.37%	−16.77%	Rpn9; eIF4G2; Pros26.4; CG13599; SMC1
* Df(3R)mbc-R1*	95A5-7;95D6-11	1302	32	49	1464	11.07%*^c^*	−20.22%	−14.75%	Rpn9; eIF4G2; Pros26.4; CG13599; SMC1
* Df(3R)Exel6202*	96D1;96D1	837	64	29	1023	18.18%*^c^*	−10.68%	−7.64%	
* Df(3R)BSC42*	98B1-2;98B3-5	1849	126	35	2171	14.83%*^c^*	−16.45%	−10.99%	Sce; btz
* Df(3R)3450*	98E3;99A6-8	1003	42	28	1143	12.25%*^c^*	−7.26%	−13.57%	Doa; Slu7; yemaα; dgt6; Slbp; stg

^a^ Difference between X NDJ of the deficiency-bearing flies *vs.* matched control.

^b^ Difference between X NDJ of the deficiency-bearing flies *vs.* average controls.

^c^ The percentage of X NDJ is significantly higher/lower than in *X/X;bubR1^D1326N^/bubR1^rev1^* females (multinomial-Poisson hierarchy model, *P* < 0.05).

### Enhancers of *bubR1* X chromosome NDJ

In the group of deficiencies that enhance the frequency of *bubR1* X NDJ, we narrowed down to a smaller genomic region or identified candidate genes for the following deficiencies: *Df(3R)D605* (97E2;98A3-4), *Df(3L)Pc-2q* (78C5-6;78E3-79A1), and *Df(3L)BSC13* (66B12-C1;66D2-4). Because the breakpoints of some of these deficiencies have not been defined molecularly, it is likely that these regions and the genes they contain cannot be accurately defined.

#### Df(3R)D605.

*Df(3R)D605* (97E2;98A3-4) enhances *bubR1* X NDJ by 17.19% when compared with its own matched control (Table 2). To identify a smaller genomic region, we tested four overlapping deficiencies [*Df(3R)ED6255* (97D2;97F1); *Df(3R)Exel6206* (97E1;97E5); *Df(3R)ED6237* (97E4;97E11) and *Df(3R)IR16* (97F1-2;98A)] and one deficiency covering completely *Df(3R)D605* [*Df(3R)ED6265* (97E2;98A7)]. Among these five deficiencies, only *Df(3R)ED6265* has a similar effect as *Df(3R)D605* on *bubR1* X NDJ, indicating that the gene responsible for the observed enhancement lies within 98A1;98A4 genomic region (Table S3).

#### Df(3L)Pc-2q.

*Df(3L)Pc-2q* (78C5-6;78E3-79A1) enhances *bubR1* X NDJ by 18.43% when compared to its own matched control (Table 2). Within the third chromosome deficiency kit, we also tested *Df(3L)ED4978* (78D5;79A2) which partially overlaps with *Df(3L)Pc-2q*. However, *Df(3L)ED4978* suppresses *bubR1* X NDJ by 6.52% when compared to its own matched control (Table 2). Thus, according to the cytogenetic map coordinates for both deficiencies, we can conclude that the enhancer gene is located in the genomic region 78C5-6;78D4, while the suppressor gene is located in the genomic region 79A1;79A2.

#### Df(3L)BSC13.

*Df(3L)BSC13* (66B12-C1;66D2-4) enhances *bubR1* X NDJ by 18.24% when compared with its own matched control (Table 2). Within the third chromosome deficiency kit, we also tested *Df(3L)ZP1* (66A17-20;66C1-5), which partially overlaps with *Df(3L)BSC13*. However, *Df(3L)ZP1* suppresses *bubR1* X NDJ (Table 2), suggesting that the enhancer gene should be in the region 66C6-66D4. Within this region, we identified *matrimony* (*mtrm* – 66C11) as a candidate gene. *mtrm* was previously identified in a screen of the major autosomes as being haploinsufficient for achiasmate segregation in *Drosophila* oocytes (Harris *et al.* 2003). We tested a null allele of *mtrm*, *mtrm^126^* (Xiang *et al.* 2007) in a *bubR1^D1326N^/bubR1^rev1^* mutant female background and observed a 13.40% increase of *bubR1* X NDJ. However, *mtrm^126^/+* females have a frequency of X NDJ of 9.45%. Thus, it is likely that the enhancement of *bubR1* X NDJ observed in *bubR1^D1326N^/bubR1^rev1^; mtrm^126^/+* females is only due to an additive effect of the frequency of NDJ displayed by *mtrm* and *bubR1* mutants alone.

### Suppressors of *bubR1* X NDJ

In the group of deficiencies that suppress the frequency of *bubR1* X NDJ, we narrowed down to a smaller genomic region or identified candidate genes for the following deficiencies: *Df(3R)ED5559* (86E11;87B11) and *Df(3L)rdgC-co2* (77A1;77D1).

#### Df(3R)ED5559.

*Df(3R)ED5559* (86E11;87B11) completely suppresses *bubR1*-induced X NDJ (Table 2). To uncover the gene responsible for the full phenotypic rescue, we tested four deficiencies that overlap *Df(3R)ED5559* [*Df(3R)ED5516* (86D8;86E13); *Df(3R)Exel8154* (86E13;86E18); *Df(3R)Exel7310* (86E18;87A1) and *Df(3R)ED5577* (86F9;87B13)]. Our results show that only *Df(3R)ED5577* has a similar effect, suggesting that the candidate gene is within the genomic region 87A2;87B11. In this region, we identified *aurora* (*aur*) as a potential candidate gene. Aur is essential for several aspects of the cell cycle and mitotic progression (Berdnik and Knoblich 2002; Marumoto *et al.* 2003; Portier *et al.* 2007). We tested two EMS *aur* alleles, *aur^1^* (hypomorphic allele) and *aur^87Ac-3^* (null allele) (Glover *et al.* 1995). However, none of these alleles showed a full rescue of *bubR1* X NDJ, suggesting that a different gene within the region is responsible for this effect (Table S3).

#### Df(3L)rdgC-co2.

*Df(3L)rdgC-co2* (77A1;77D1) suppresses *bubR1* X NDJ by 14.68% when compared with its own matched control (Table 2). We identified two other overlapping deficiencies that also suppress *bubR1* X NDJ: *Df(3L)ED4858* (76D3;77C1) and *Df(3L)Exel6136* (77B2;77C6), suggesting that the gene responsible is within the genomic region 77B2;77C1 (Table 3). In this genomic region, we identified *polo* (77B2-77B3) as a candidate gene, so we tested three *polo* mutant alleles (*polo^1^*, *polo^2^*, *and polo^9^*). Our results show that all three alleles suppress *bubR1* X NDJ and the level of suppression increases with stronger *polo* alleles (Table 3). *polo^1^* is a weak hypomorph allele with an EMS-induced point mutation in the kinase domain and is hemizygous viable (Sunkel and Glover 1988). *polo^9^* allele has a *P*-element insertion that reduces Polo levels, and is homozygous lethal at third-instar larvae (Donaldson *et al.* 2001). *polo^2^* was induced by P-M hybrid dysgenesis and is consider as the strongest *polo* allele (C.E. Sunkel, R.E. Karess, and D.M. Glover, unpublished results).

**Table 3:** NDJ of *Df(3L)rdgC-co2*, its overlapping deficiencies, and mutant alleles

Maternal Genotype	Cytogenetic Breakpoints	Normal ProgenyX/XY and X/O	Exceptional Progeny		Total Adjusted Progeny	X NDJ
			XX/O	O/XY		
*bubR1^D1326N^/bubR1^rev1^*		737	102	34	1009	26.96%
*bubR1^D1326N^/bubR1^rev1^;Df(3L)rdgC-co2/+*	77A1;77D1	686	37	11	782	12.28%*^a^*
*bubR1^D1326N^/bubR1^rev1^;Df(3L)ED4858/+*	76D3;77C1	566	23	13	638	11.29%*^a^*
*bubR1^D1326N^/bubR1^rev1^;Df(3L)Exel6136/+*	77B2;77C6	1996	9	5	2024	1.38%*^a^*
*bubR1^D1326N^/bubR1^rev1^;polo^1^/+*		438	35	17	542	19.19%*^a^*
*bubR1^D1326N^/bubR1^rev1^;polo^2^/+*		1612	32	25	1726	6.60%*^a^*
*bubR1^D1326N^/bubR1^rev1^;polo^9^/+*		979	36	21	1093	10.43%*^a^*

^a^ The percentage of X NDJ is significantly lower than in *X/X;bubR1^D1326N^/bubR1^rev1^* females (multinomial-Poisson hierarchy model, *P* < 0.05).

During meiosis, *CDC5*, the yeast *polo* homolog, is required to phosphorylate and remove meiotic cohesin from chromosome arms (Lee and Amon 2003), to form chiasmata (Clyne *et al.* 2003), to co-orient sister kinetochores and to cosegregate sister centromeres at meiosis I (Clyne *et al.* 2003; Lee and Amon 2003). In *D. melanogaster* meiosis, Polo is involved in the timing of meiotic prophase I entry, in the restriction of meiosis to the oocyte and in the initiation/maintenance of SC (Mirouse *et al.* 2006). At later stage, Polo is required to activate Twine, a germline-specific form of the Cdc25 phosphatase, that initiates the chain of events leading to GVBD and prometaphase I progression (Xiang *et al.* 2007). At meiosis II, Polo is essential to phosphorylate and remove MEI-S332, the Shugoshin homolog, and to allow sister chromatid segregation (Clarke *et al.* 2005).

**Figure 1:**
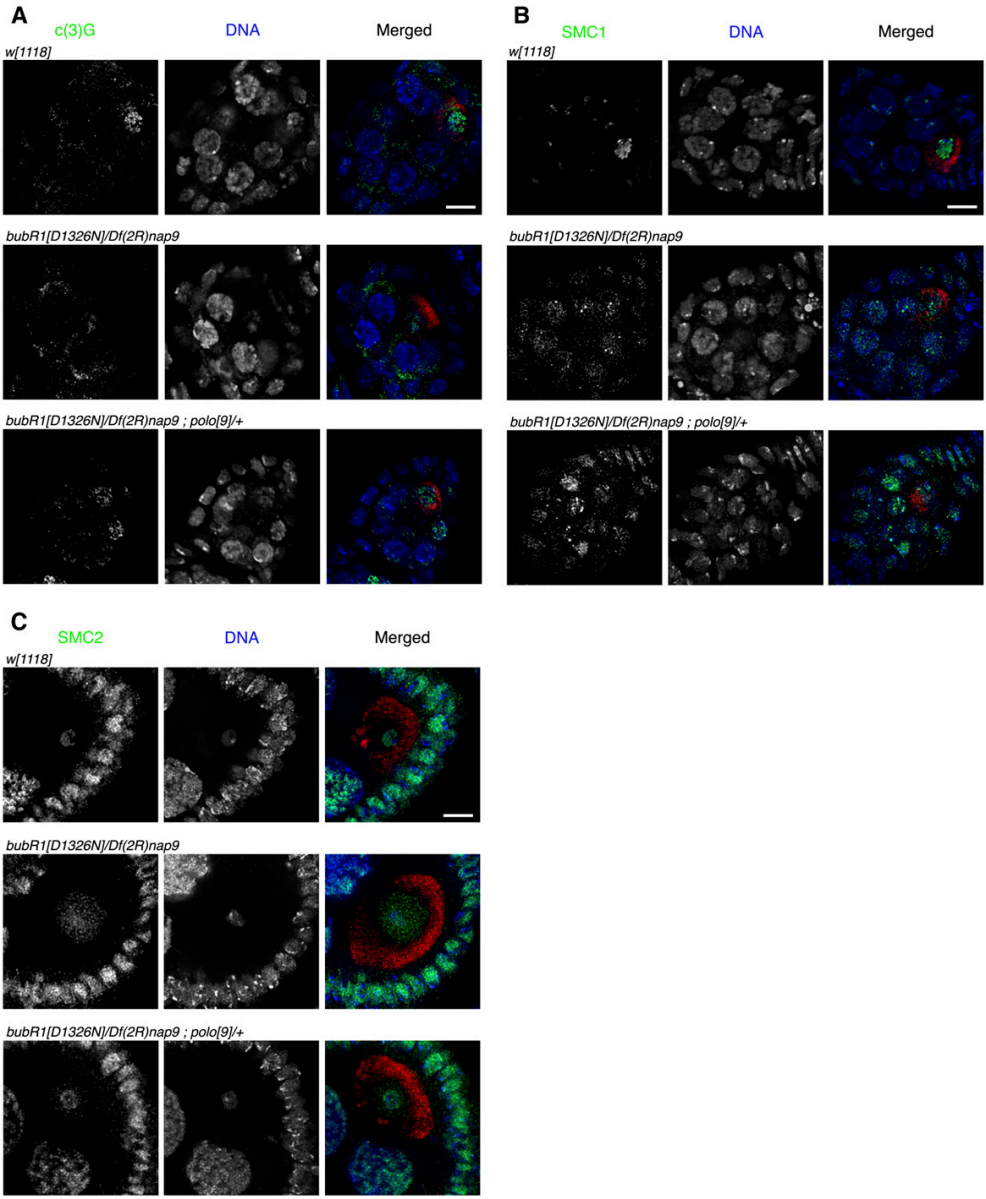
Polo is a suppressor of *bubR1* X NDJ. (A) Structure of SC in wild-type, in *bubR1* mutant and in *bubR1;polo* mutant in region 3 of the germarium. C(3)G is in green, DNA is in blue, and Orb (as oocyte marker) is in red. Decreasing *polo* dosage in a *bubR1* genetic background rescues the maintenance of the SC in the oocyte nucleus comparing to *bubR1* mutant. (B) Sister chromatid cohesin subunit SMC1 in wild-type, in *bubR1* mutant and in *bubR1;polo* mutant in region 3 of the germarium. SMC1 is in green, DNA is in blue, and Orb is in red. Decreasing *polo* dosage in *bubR1* genetic background rescues the maintenance of the sister chromatid cohesion in the oocyte nucleus comparing to *bubR1* mutant. (C) Condensin subunit SMC2 in wild-type, in *bubR1* mutant and in *bubR1;polo* mutant at the pachytene/diplotene transition (stages 5-7). SMC2 is in green, DNA is in blue, and Orb is in red. Decreasing *polo* dosage in *bubR1* genetic background rescues the loading of condensin subunit at stages 5–7 in the oocyte nucleus during chiasmata formation comparing to *bubR1* mutant oocyte. Scale bars = 10 μm.

To further investigate at the molecular level the nature of the decrease in X NDJ, we first analyzed whether reducing Polo dosage in *bubR1* mutant females restores in part, the meiotic prophase I phenotypes associated with the *bubR1* mutation. Because BubR1 and Polo appear to have opposite effects during early stages of meiotic prophase I, with Polo controlling meiotic entry and SC assembly and BubR1 controlling meiotic prophase I progression and SC disassembly, we analyzed the nature of the SC and sister chromatid cohesion in *bubR1^D1326N^/Df(2R)nap9*;*polo^9^/+* mutant combinations in region 3 of the germarium (Figure 1). Interestingly, while mutations in *bubR1* result in premature disassembly of the SC, in the *bubR1^D1326N^/Df(2R)nap9*;*polo^9^/+* we observed the maintenance of the SC protein, C(3)G, and the sister chromatid cohesin subunit, SMC1 (Figure 1, A and B). This suggests that decreasing Polo dosage in *bubR1* mutant ovarioles delays sufficiently prophase I entry and/or progression to allow a more accurate prophase I. In addition, we investigated if a reduction of Polo dosage in *bubR1* mutant females allows a higher efficiency in the specific replacement of cohesin complex by condensin complex during chiasmata formation (Yu and Koshland 2005) and SC disassembly (Ivanovska *et al.* 2005; Resnick *et al.* 2009). Thus, we analyzed the localization of SMC2 condensin subunit at stage 5-7, which corresponds to the pachytene-diplotene transition. In wild-type oocytes, condensin subunit SMC2 is gradually loaded on the bivalent to fully cover the karyosome by stage 5-7 (Figure 1C). However, in *bubR1* mutant oocytes, SMC2 localizes within the nuclear space rather than being bound to the karyosome (Figure 1C). Thus, in addition to the decrease in the maintenance of the SC and of the sister chromatid cohesion, mutations in *bubR1* also affect the changes in bivalent configuration at diplotene. Interestingly, decreasing Polo dosage restores condensin loading and bivalent modifications in *bubR1* mutant oocytes. Taken together, these results suggest that the decreased X NDJ induced by a reduction of Polo dosage in *bubR1* mutant oocytes occurs through a Polo-dependent mechanism during the initial stages of meiosis.

### Deficiency causing sterility in *bubR1^D1326N^/bubR1^rev1^* females

*Df(3L)ED4674* (73B5;73E5) causes sterility in *bubR1^D1326N^/bubR1^rev1^* mutant females, suggesting a strong enhancement of the *bubR1* meiotic phenotype. To narrow down the genomic region of interest, we tested four overlapping deficiencies [*Df(3L)BSC561* (73A2;73C1); *Df(3L)Exel9004* (73D1;73D5); *Df(3L)Exel7253* (73D5;73E4) and *Df(3L)BSC414* (73E1;74C3)]. Among these four deficiencies, our results show that only *Df(3L)BSC561* causes sterility in *bubR1^D1326N^/bubR1^rev1^* mutant females, indicating the presence of a dosage-sensitive gene within the genomic region 73B5-73C1. Within this region, we found two potential candidate genes, *Baldspot* and *Lasp*, which have an essential function during spermatogenesis (Jung *et al.* 2007; Lee *et al.* 2008).

### Identification of two deficiencies affecting somatic BubR1 kinase activity

It was previously shown that different domains of BubR1 have different functions, with the KEN box being essential for SAC activity but the kinase domain being required only for spindle formation during prometaphase (Elowe *et al.* 2010). Mutations in BubR1 kinase domain give rise to viable adults lacking any visible phenotype, and mutant cells are able to properly segregate their genetic material during somatic cell division (Malmanche *et al.* 2007; Rahmani *et al.* 2009). Interestingly, we identified two deficiencies that induce an embryonic lethality in the *bubR1* genetic background indicating that a decrease in some components of the cell cycle network can impair cell cycle progression and zygote development in a BubR1 kinase dead context.

#### Df(3L)BSC10.

**Figure 2:**
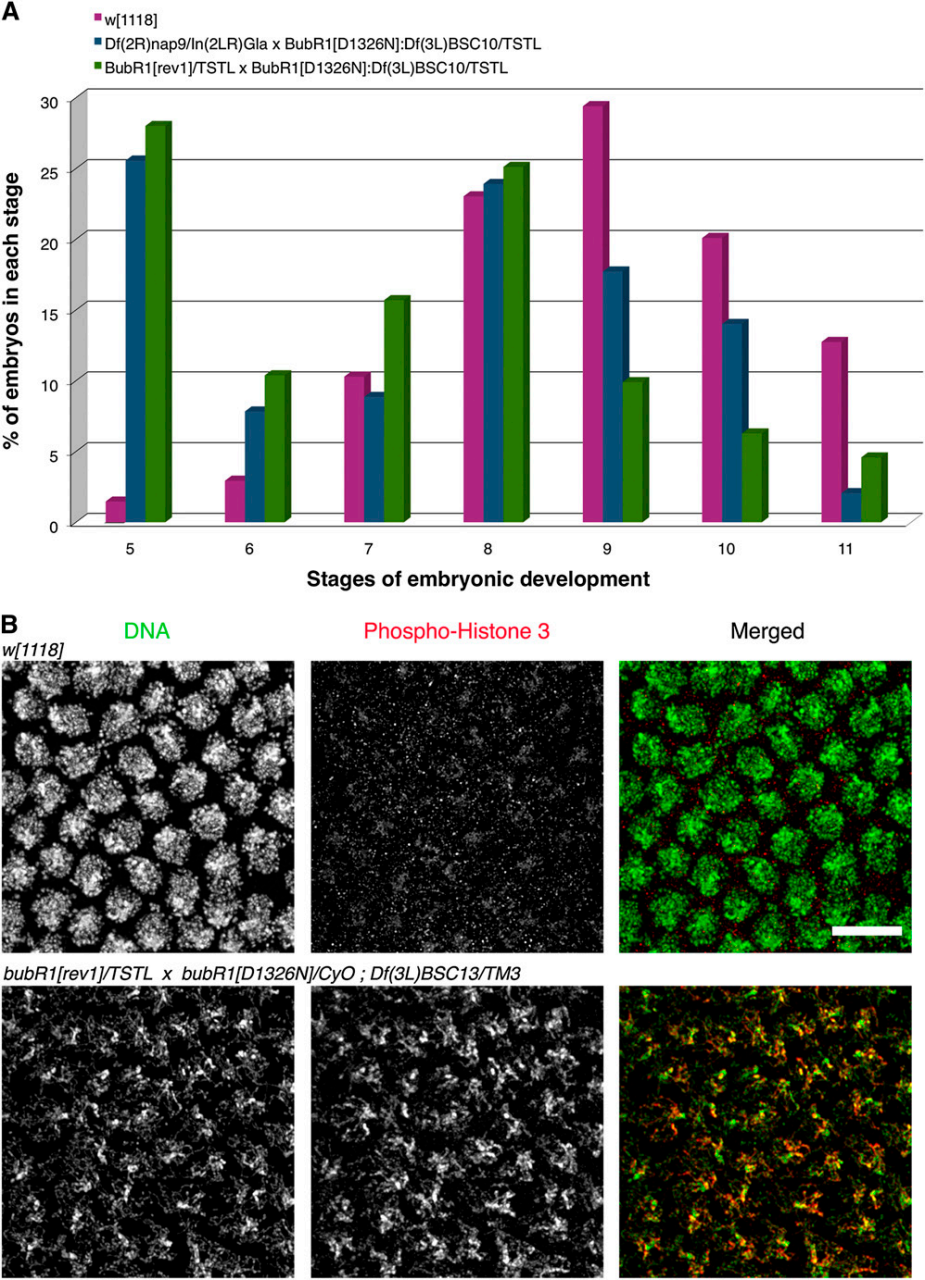
BubR1 kinase activity is essential during somatic cell cycle (A) The developmental arrest induced by *Df(3L)BSC10* in BubR1 kinase dead embryos takes place after the syncitial division and prevents the initialization of gastrulation. (B) Embryonic phenotype at stage 5 after 250 μM colchicine treatment in wild-type and progeny from a cross made between *bubR1^rev1^/TSTL* females to *bubR1^D1326N^/CyO;Df(3L)BSC10/TM3* males. Phospho-Histone 3 is in red and DAPI is in green. In contrast to wild-type embryos, replicated chromosomes in mutant embryos after colchicine treatment appear condensed and the presence of phospho-histone 3 epitope is detected. Scale bars = 10μm.

*Df(3L)BSC10* deletes the genomic region 69D4-5;69F5-7. First, we characterized the embryonic stage at which the development of *bubR1^D1326N^/bubR1^rev1^;Df(3L)BSC10/+* embryos fail to proceed. For this, we analyzed staged embryonic collections from 0-2 hr that were aged for 3 hr before fixation. Our results showed a developmental arrest at stage 5 that corresponds to the point when the syncytial cell cycle 13 ends and cellularization and gastrulation initiates (Figure 2A). Thus, we asked if the mutant embryos failed to undergo gastrulation due to a modified cytoskeleton network. For this, embryos were collected as before, but they were treated with colchicine prior to fixation, to depolymerize microtubules. In wild-type embryos, colchicine treatment does not affect the overall nuclear structure, and the replicated chromosomes remain decondensed (Figure 2B). However, in the mutant embryos, we observed the condensation of interphase chromatin after colchicine treatment. Moreover, chromosome condensation is associated with the presence of the phospho-H3 epitope that is normally only detected in dividing nuclei (Figure 2B). To identify candidate genes within this deficiency, we tested overlapping deficiencies covering the *Df(3L)BSC10* deleted region [*Df(3L)iro-2* (69B1-5;69D1-6); *Df(3L)Exel6117* (69D1;69E2), *Df(3L)E44* (69D2;69E3-5) and *Df(3L)ED4486* (69C4;69F6)]. However, none of the deficiencies tested induce an embryonic lethal phenotype, suggesting that the candidate gene is within the genomic region 69F6;69F7. Within this small region, we found *RpS4* (*Ribosomal protein S4*), a gene involved in mitotic spindle elongation and organization (Goshima *et al.* 2007). However, we cannot exclude the possibility of *Df(3L)BSC10* having another unknown chromosomal aberration.

#### Df(3R)BSC56.

*Df(3R)BSC56* (94E1-2;94F1-2) also induces a developmental arrest of *bubR1^D1326N^/bubR1^rev1^;Df(3R)BSC56/+* mutant embryos. We used five overlapping deficiencies covering the *Df(3R)BSC56* deleted region to identify candidate genes [*Df(3R)BSC55* (94D2-10;94E1-6), *Df(3R)Exel6193* (94D3;94E4), *Df(3R)ED6103* (94D3;94E9), *Df(3R)Exel6274* (94E4;94E11) and *Df(3R)Exel6194* (94F1;95A4)]. Among these deficiencies, only *Df(3R)Exel6274* induces an embryonic lethality, suggesting the presence of the candidate gene within the genomic region 94E9;94E11, defined between the breakpoint of *Df(3R)ED6103* and *Df(3R)Exel6274*. Within this region, we identified *cdc16* (94E9) as a potential candidate gene. Cdc16/Apc6 is a tetratricopeptide repeat (TPR) subunit of APC/C, essential for G2 progression. *cdc16^RNAi^* animals die as P5(i) early pupae and *cdc16^RNAi^* cells show mitotic phenotypes, including high mitotic index and overcondensed chromosomes in a metaphase-like arrest and depletion of Cdc16 also affects cyclin B degradation (Pal *et al.* 2007). We therefore tested a *cdc16* allele (*cdc16^MB09129^*) that has a transposable element insertion in an intron of *cdc16*. However, this allele does not induce the lethality observed with *Df(3R)BSC56*, suggesting that either *cdc16* is not the relevant gene or the allele we tested, which has not been extensively characterized, still produces sufficient active protein that allow embryonic development.

## Discussion

We have shown previously that BubR1 kinase activity is essential during meiotic prophase I progression to ensure the correct timing of SC disassemble and chromosome NDJ in *D. melanogaster* female (Malmanche *et al.* 2007). Given the frequency of chromosome NDJ observed in a dose-sensitive manner in *bubR1^D1326N^ trans*-heterozygotes, we decided to perform a genetic screen to identify third-chromosome haploinsufficient synthetic modifiers of the NDJ phenotype. We could test 65 of the 96 deficiencies that are part of the third chromosome deficiency kit generated by the Bloomington *Drosophila* Stock Center. We could not test all of them due to the presence of haplolethal and haplosterile loci (Marygold *et al.* 2007) in some deficiencies. Within the 60% of the euchromatin screened, we identified enhancers and suppressors of *bubR1* X NDJ. Furthermore, we show that Polo kinase acts as a strong suppressor of female *bubR1* X NDJ by delaying meiotic prophase I progression. Interestingly, we also found deficiencies that cause synthetic lethality or affect the fertility of the mutant females. Thus, this screen proved to be highly sensitive for the identification of modifiers of the BubR1 kinase activity.

Ideally, this genetic screen should test simultaneously all mutant genetic backgrounds to minimize variables, such as temperature, humidity and food. However, because the genotype used to test NDJ only accounts for 1/6 of the total female F2 generation, all the third-chromosome deficiencies could not be tested at the same time. Therefore, we performed 16 different experiments. To classify the deficiencies as enhancer, suppressor, or having no effect, we took into account the variation in X NDJ observed in the control genotype and then performed a statistical analysis using the multinomial-Poisson hierarchy model (Zeng *et al.* 2010) with a 95% confidence interval.

From the 65 deficiencies tested, we identified six deficiencies that enhance and 18 deficiencies that suppress female *bubR1* X NDJ. From these 24 positive hits, we narrowed down the genomic region of interest using overlapping deficiencies for three enhancers [*Df(3R)D605*, *Df(3L)Pc-2q* and *Df(3L)BSC13*], and two suppressors [*Df(3R)ED5559* and *Df(3L)rdgC-co2*] to identify the genes responsible for the modification of the X NDJ frequency. We identified the gene responsible for modification of X NDJ for two deficiencies: *Df(3L)BSC13* and *Df(3L)rdgC-co2*. Further studies are needed to identify among the subset of candidate genes, those responsible for the modification of the female X NDJ for the other deficiencies.

Despite the difficulties in identifying the gene involved in the process of X NDJ for every positive interaction, our genetic strategy allowed us to identify Polo kinase as a suppressor of *bubR1* X NDJ. Our previous results (Malmanche *et al.* 2007) illustrate an essential requirement for BubR1 kinase activity in the timing of the early stages of meiotic prophase I, suggesting that BubR1 and Polo have opposite functions in early prophase I. BubR1 appears to slow down progression by maintaining the SC in place, whereas Polo accelerates the process by driving prophase I forward. Through indirect immunofluorescence, we confirmed that Polo dosage antagonizes BubR1 function during the early stages of meiotic prophase I, first by increasing SC and sister chromatin cohesion maintenance, and second by allowing a higher efficiency of bivalent reorganization during karyosome formation, as observed by the loading of the condensin subunit SMC2 at pachytene-diplotene transition.

Our genetic screen also allowed the identification of two deficiencies that impair BubR1 kinase activity in somatic cells and zygotic development. It has been recently shown that BubR1 kinase activity is not essential for accurate somatic chromosome segregation and animal development, because BubR1 kinase dead homozygous adult flies lack any obvious phenotype and are recovered in a normal Mendelian proportion (Malmanche *et al.* 2007; Elowe *et al.* 2010). Nonetheless, although these results strongly suggest that BubR1 kinase activity is not essential for somatic development, our findings indicate that subtle modifications in the dosage of other key components of the cell cycle machinery can trigger an essential requirement for BubR1 kinase activity.

Taken together, our results suggest that the genetic screen strategy presented above is highly efficient in recovering genes that interact with BubR1 kinase function. Our results allowed us to identify deficiencies showing genetic interactions, spanning from full recovery of X NDJ to sterility, the strongest effect expected for genes positively involved in BubR1 kinase activity. In addition, the screen also revealed an unexpected result, because it identified deficiencies that impaired zygotic development of BubR1 kinase dead embryos, suggesting a somatic phenotype. Further genetic and cytological experiments will allow the identification of genes responsible for the effect observed in the characterized deficiencies, allowing the build up of a genetic map of essential genes involved in BubR1 kinase function.

## Supplementary Material

Supporting Information
