# Supplementary material for: polo Is Identified as a Suppressor of bubR1 Nondisjunction in a Deficiency Screen of the Third Chromosome in Drosophila melanogaster
Source: G3 (Bethesda). 2011 Jul 1;1(2):161–9. doi: 10.1534/g3.111.000265 (PMC3276128; doi:10.1534/g3.111.000265)
Supplement: Supporting Information [file supp_1_2_161__index.html]

Supporting Information 

# *polo* Is Identified as a Suppressor of *bubR1* Nondisjunction in a Deficiency Screen of the Third Chromosome in *Drosophila melanogaster*

## Supporting Information for Sousa-Guimaraes *et al.*, 2011

**Files in this Data Supplement:**

- Supporting Information - Tables S1-S3 (PDF, 212 KB)
- Table S1 - Third chromosome deficiencies that do not affect *bubR1* X NDJ (PDF, 76 KB)
- Table S2 - Complementation tests of the deficiencies that affect *bubR1* X NDJ (PDF, 96 KB)
- Table S3 - Refining the mapping of *bubR1* modifiers (PDF, 56 KB)
