# Supplementary material for: polo Is Identified as a Suppressor of bubR1 Nondisjunction in a Deficiency Screen of the Third Chromosome in Drosophila melanogaster
Source: G3 (Bethesda). 2011 Jul 1;1(2):161–9. doi: 10.1534/g3.111.000265 (PMC3276128; doi:10.1534/g3.111.000265)
Supplement: Supporting Information [file supp_1.2.161_000265SIf.pdf]

Table S1 Third chromosome deficiencies that do not affect *bubR1* X NDJ

Deficiency name	Cytogenetic breakpoints	Normal progeny	Exceptional progeny		Total adjusted progeny	X NDJ	d With matched control[(a)]	d With average control[(b)]
		X/XY & X/O	XX/O	O/XY				
Df(3L)emc-E12	61A;61D3	258	56	6	382	32.46%	-2.74%	6.64%
Df(3L)Ar14-8	61C5-8;62A8	993	126	28	1301	23.67%*	4.17%	-2.15%
Df(3L)Aprt-1	62A10-B1;62D2-5	778	107	36	1064	26.88%	2.34%	1.06%
Df(3L)R-G7	62B7;62E5-6	402	82	15	596	32.55%	-0.23%	6.73%
Df(3L)GN34	63E6-9;64A8-9	332	26	10	404	17.82%	-5.17%	-8.00%
Df(3L)ED4342	64A12;64B12	756	151	31	1120	32.50%	3.64%	6.68%
Df(3L)XDI98	65A2;65E1	837	130	21	1139	26.51%	1.51%	0.69%
Df(3L)BSC27	65D4-5;65E4-6	895	107	30	1169	23.44%	-1.10%	-2.38%
Df(3L)BSC33	65E10-F1;65F2-6	959	196	12	1375	30.25%*	5.25%	4.43%
Df(3L)66C-G28	66B8-9;66C9-10	754	136	16	1058	28.73%*	-6.47%	2.91%
Df(3L)h-i22	66D10-11;66E1-2	459	51	16	593	22.60%	-0.39%	-3.22%
Df(3L)Scf-R6	66E1-6;66F1-6	1071	189	30	1509	29.03%	-2.25%	3.21%
Df(3L)BSC35	66F1-2;67B2-3	142	27	7	210	32.38%	-0.40%	6.56%
Df(3L)eyg[C1]	69A4-5;69D4-6	664	107	20	918	27.67%	0.26%	1.85%
Df(3L)BSC12	69F6-70A1;70A1-2	584	86	19	794	26.45%	3.46%	0.63%
Df(3L)fz-GF3b	70C1-2;70D4-5	75	10	5	105	28.57%	0.51%	2.75%
Df(3L)fz-M21	70D2-3;71E4-5	1368	154	38	1752	21.92%	-0.84%	-3.90%
Df(3L)Cat	75B4-7;75E2	354	29	19	450	21.33%	-3.20%	-4.49%
Df(3L)fz2	75F10-11;76A1-5	537	41	15	649	17.26%	0.66%	-8.56%
Df(3L)ED4782	75F2;76A1	911	101	14	1141	20.16%	-4.38%	-5.66%
Df(3L)BSC20	76A7-B1;76B4-5	146	47	5	250	41.60%	6.40%	15.78%
Df(3L)ri-Xt1	77E2-4;78A2-4	801	109	18	1055	24.08%*	4.57%	-1.74%
Df(3L)HD1	79D3-E1;79F3-6	1209	130	41	1551	22.05%	-1.31%	-3.77%
Df(3L)BSC21	79E5-F1;80A2-3	171	25	5	231	25.97%	-2.09%	0.15%
Df(3R)ME15	81F3-6;82F5-7	459	83	21	667	31.18%	3.12%	5.36%
Df(3R)3-4	82F3-4;82F10-11	570	114	25	848	32.78%	4.72%	6.96%
Df(3R)e1025-14	82F8-10;83A1-3	748	99	26	998	25.05%	0.51%	-0.77%

Df(3R)BSC47	83B7-C1;83C6-D1	520	64	19	686	24.20%	-3.21%	-1.62%
Df(3R)roe	84A6-B1;84D4-9	926	120	34	1234	24.96%	-3.90%	-0.86%
Df(3R)Cha7	90F1-4;91F5	1605	133	30	1931	16.88%	-2.62%	-8.94%
Df(3R)BSC43	92F7-93A1;93B3-6	549	77	11	725	24.28%	4.77%	-1.54%
Df(3R)e-N19	93B;94	929	174	40	1357	31.54%	0.26%	5.72%
Df(3R)ED6093	94A2;94C4	864	101	33	1132	23.67%	-5.19%	-2.15%
Df(3R)Exel6192	94B11;94D3	326	24	21	416	21.63%	-5.78%	-4.19%
Df(3R)crb-F89-4	95D7-11;95F15	1241	149	28	1595	22.19%	-2.34%	-3.63%
Df(3R)crb87-5	95F6-8;96A18-20	1232	113	34	1526	19.27%	-0.24%	-6.55%
Df(3R)Espl3	96F1;97B1	356	105	5	576	38.19%	2.99%	12.37%
Df(3R)Tl-P	97A;98A1-2	260	52	4	372	30.11%	-1.17%	4.29%

*The percentage of X NDJ is significantly higher/lower than in *X/X ; bubR1*^{D1326N}*/bubR1*^{rev1} females (multinomial-Poisson hierarchy model, $P < 0.05$).
[a] Difference between X NDJ of the deficiency-bearing flies *vs.* matched control.
[b] Difference between X NDJ of the deficiency-bearing flies *vs.* average controls.

Table S2 Complementation tests of the deficiencies that affect *bubR1* X NDJ

Deficiency name used in the screen	Cytogenetic breakpoints	Cross to	Deficiency used for the complementation test	Cytogenetic breakpoints	Result of the test
Df(3L)BSC23	62E8;63B5-6	x	*Df(3L)Exel6091*	62E8;62F5	lethal
		x	*Df(3L)ED4288*	63A6;63B7	lethal
Df(3L)pbl-X1	65F6;66B7-8	x	*Df(3L)ZP1*	66A17-20;66C1-5	lethal
Df(3L)ZP1	66A17-20;66C1-5	x	*Df(3L)66C-G28*	66B8-9;66C9-10	lethal
Df(3L)BSC13	66B12-C1;66D2-4	x	*Df(3L)ZP1*	66A17-20;66C1-5	lethal
Df(3L)BSC10	69D4-5;69F5-7	x	*Df(3L)eyg[C1]*	69A4-5;69D4-6	lethal
		x	*Df(3L)iro-2*	69B1-5;69D1-6	lethal
		x	*Df(3L)ED4486*	69C4;69F6	lethal
		x	*Df(3L)Exel6117*	69D1;69E2	lethal
		x	*Df(3L)E44*	69D2;69E3-5	lethal
		x	*Syx13[01470]*	69F6	viable
		x	*Df(3L)BSC12*	69F6-70A1;70A1-2	viable
Df(3L)ED4674	73B5;73E5	x	*Df(3L)BSC561*	73A2;73C1	lethal
		x	*Df(3L)Exel6130*	73B5;73D1	lethal
Df(3L)BSC8	74D3-75A1;75B2-5	x	*Df(3L)BSC415*	74A5;75A4	lethal
		x	*Df(3L)BSC775*	75A2;75E4	lethal
Df(3L)ED4858	76D3;77C1	x	*Df(3L)Exel6136*	77B2;77C6	lethal
Df(3L)rdgC-co2	77A1;77D1	x	*Df(3L)ED4858*	76D3;77C1	lethal
		x	*Df(3L)ri-79c*	77B-C;77F-78A	lethal
		x	*Df(3L)Exel6136*	77B2;77C6	lethal
Df(3L)Pc-2q	78C5-6;78E3-79A1	x	*Df(3L)ME107*	77F3;78C8-9	viable
		x	*Df(3L)Exel9065*	78D4;78D5	lethal
		x	*Df(3L)Exel9066*	78D5;78D6	lethal
		x	*Df(3L)ED4978*	78D5;79A2	lethal
Df(3L)ED4978	78D5;79A2	x	*Df(3L)Pc-2q*	78C5-6;78E3-79A1	lethal
		x	*Df(3L)Exel9065*	78D4;78D5	viable
		x	*Df(3L)Exel9066*	78D5;78D6	lethal
Df(3L)Ten-m-AL29	79C1-3;79E3-8	x	*Df(3L)ED230*	79C2;80A4	lethal
		x	*Df(3L)HD1*	79D3-E01;79F3-6	lethal
		x	*Df(3L)Exel6138*	79D3;79E3	lethal
		x	*Df(3L)BSC21*	79E5-F1;80A2-3	lethal
Df(3R)ED5177	83B4;83B6	x	*Df(3R)Exel6144*	83A6;83B6	lethal
		x	*Df(3R)BSC549*	83A6;83B6	lethal
Df(3R)Tpl10	83C1-2;84B1-2, 83D4-5;84A4-5;98F1-2	x	*Df(3R)BSC47*	83B7-C1;83C6-D1	lethal
		x	*Df(3R)ED5197*	83B7;83D2	viable
		x	*Df(3R)Exel7284*	83C4;83D2	viable
		x	*Df(3R)Tpl6*	83D1-2;84A4-5	lethal
		x	*Df(3R)WIN11*	83E1-2;84A5	lethal
		x	*Df(3R)Scr*	84A1-2;84B1-2	lethal
		x	*Df(3R)Antp17*	84A5;84D9	lethal
Df(3R)GB104	85D12;85E10	x	*Df(3R)BSC476*	85D16;85D24	lethal
		x	*Df(3R)Exel6153*	85D19;85E1	lethal
Df(3R)ED5559	86E11;87B11	x	*Df(3R)ED5577*	86F9;87B13	lethal

		x	*Df(3R)Exel7313*	87A9;87B5	lethal
Df(3R)sbd105	88F9-89A1;89B9-10	x	*Df(3R)Exel7327*	89A8;89B1	viable
Df(3R)ED5780	89E11;90C1	x	*Df(3R)Exel6176*	89E11;89F1	lethal
		x	*Df(3R)BSC790*	90B6;90E2	lethal
Df(3R)ED5942	91F12;92B3	x	*Df(3R)BSC475*	91F12;92B4	lethal
Df(3R)BSC56	94E1-2;94F1-2	x	*Df(3R)BSC55*	94D2-10;94E1-6	lethal
		x	*Df(3R)Exel6193*	94D3;94E4	lethal
		x	*Df(3R)ED6103*	94D3;94E9	lethal
		x	*Df(3R)Exel6274*	94E4;94E11	lethal
		x	*Df(3R)Exel6194*	94F1;95A4	viable
Df(3R)mbc-R1	95A5-7;95D6-11	x	*Df(3R)Exel6194*	94F1;95A4	lethal
		x	*Df(3R)mbc-30*	95A5-7;95C10-11	lethal
		x	*Df(3R)Exel9014*	95B1;95D1	lethal
		x	*Df(3R)Exel6196*	95C12;95D8	lethal
		x	*Df(3R)crb-F89-4*	95D7-D11;95F15	lethal
		x	*Df(3R)Exel6197*	95D8;95E5	lethal
Df(3R)Exel6202	96D1;96D1	x	*Df(3R)BSC849*	96D1;96E2	lethal
		x	*Df(3R)BSC522*	96D1;96E3	lethal
Df(3R)D605	97E3;98A5	x	*Df(3R)ED6255*	97D2;97F1	lethal
		x	*Df(3R)Exel6206*	97E1;97E5	lethal
		x	*Df(3R)ED6265*	97E2;98A7	lethal
		x	*Df(3R)ED6237*	97E4;97E11	lethal
		x	*Df(3R)IR16*	97F1-2;98A	lethal
Df(3R)BSC42	98B1-2;98B3-5	x	*Df(3R)BSC498*	98A4;98B5	lethal
		x	*Df(3R)BSC499*	98A14;98B5	lethal
Df(3R)3450	98E3;99A6-8	x	*Df(3R)Exel6210*	98E1;98F5	lethal
		x	*Df(3R)ED6310*	98F12;99B2	lethal
		x	*Df(3R)ED6316*	99A5;99C1	lethal

lethal – the two deficiencies do not complement each other
viable – the two deficiencies complement each other

Table S3 Refining the mapping of *bubR1* modifiers

Female genotype	Breakpoint	Normal progeny	Exceptional progeny		Total adjusted progeny	X NDJ
		X/XY & X/O	XX/O	O/XY		
		Df(3R)D605 –enhancer				
Df(3R)D605/+		2033	1	0	2035	0.10%
bubR1^{D1326N}*/bubR1*^{rev1}		3271	194	156	3971	17.63%
bubR1^{D1326N}*/bubR1*^{rev1} *; Df(3R)D605/+*	97E2;98A3-4	1010	213	26	1488	32.12%*
bubR1^{D1326N}*/bubR1*^{rev1} *; Df(3R)ED6255/+*	97D2;97F1	2720	157	61	3156	13.81%
bubR1^{D1326N}*/bubR1*^{rev1} *; Df(3R)Exel6206/+*	97E1;97E5	1233	107	26	1499	17.75%
bubR1^{D1326N}*/bubR1*^{rev1} *; Df(3R)ED6265/+*	97E2;98A7	990	216	118	1658	40.29%*
bubR1^{D1326N}*/bubR1*^{rev1} *; Df(3R)ED6237/+*	97E4;97E11	2648	214	98	3272	19.07%
bubR1^{D1326N}*/bubR1*^{rev1} *; Df(3R)IR16/+*	97F1-2;98A	1934	222	80	2538	23.80%
		Df(3R)ED5559 – suppressor				
bubR1^{D1326N}*/bubR1*^{rev1}		1452	149	48	1846	21.34%
bubR1^{D1326N}*/bubR1*^{rev1} *; Df(3R)ED5559/+*	86E11;87B11	270	0	0	270	0.00%*
bubR1^{D1326N}*/bubR1*^{rev1} *; Df(3R)ED5516/+*	86D8;86E13	651	61	19	811	19.73%
bubR1^{D1326N}*/bubR1*^{rev1} *; Df(3R)Exel8154/+*	86E13;86E18	582	52	16	718	18.94%
bubR1^{D1326N}*/bubR1*^{rev1} *; Df(3R)Exel7310/+*	86E18;87A1	863	85	21	1075	19.72%
bubR1^{D1326N}*/bubR1*^{rev1} *; Df(3R)ED5577/+*	86F9;87B13	1506	0	1	1508	0.13%*
bubR1^{D1326N}*/bubR1*^{rev1} *; aur*^{1}*/+*		1562	160	28	1938	19.40%
bubR1^{D1326N}*/bubR1*^{rev1} *; aur*$^{87Ac-3}$*/+*		1302	119	28	1596	18.42%

*The percentage of X NDJ is significantly higher/lower than in *X/X;bubR1*^{D1326N}*/bubR1*^{rev1} females (multinomial-Poisson hierarchy model, $P < 0.05$).
