# Supplementary material for: polo Is Identified as a Suppressor of bubR1 Nondisjunction in a Deficiency Screen of the Third Chromosome in Drosophila melanogaster
Source: G3 (Bethesda). 2011 Jul 1;1(2):161–9. doi: 10.1534/g3.111.000265 (PMC3276128; doi:10.1534/g3.111.000265)
Supplement: Supporting Information [file supp_1.2.161_TableS1.pdf]

Table S1 Third chromosome deficiencies that do not affect *bubR1* X NDJ

Deficiency name	Cytogenetic breakpoints	Normal progeny	Exceptional progeny		Total adjusted progeny	X NDJ	d With matched control[a]	d With average control[b]
		X/XY & X/O	XX/O	O/XY				
Df(3L)emc-E12	61A;61D3	258	56	6	382	32.46%	-2.74%	6.64%
Df(3L)Ar14-8	61C5-8;62A8	993	126	28	1301	23.67%*	4.17%	-2.15%
Df(3L)Aprt-1	62A10-B1;62D2-5	778	107	36	1064	26.88%	2.34%	1.06%
Df(3L)R-G7	62B7;62E5-6	402	82	15	596	32.55%	-0.23%	6.73%
Df(3L)GN34	63E6-9;64A8-9	332	26	10	404	17.82%	-5.17%	-8.00%
Df(3L)ED4342	64A12;64B12	756	151	31	1120	32.50%	3.64%	6.68%
Df(3L)XDI98	65A2;65E1	837	130	21	1139	26.51%	1.51%	0.69%
Df(3L)BSC27	65D4-5;65E4-6	895	107	30	1169	23.44%	-1.10%	-2.38%
Df(3L)BSC33	65E10-F1;65F2-6	959	196	12	1375	30.25%*	5.25%	4.43%
Df(3L)66C-G28	66B8-9;66C9-10	754	136	16	1058	28.73%*	-6.47%	2.91%
Df(3L)h-i22	66D10-11;66E1-2	459	51	16	593	22.60%	-0.39%	-3.22%
Df(3L)Scf-R6	66E1-6;66F1-6	1071	189	30	1509	29.03%	-2.25%	3.21%
Df(3L)BSC35	66F1-2;67B2-3	142	27	7	210	32.38%	-0.40%	6.56%
Df(3L)eyg[C1]	69A4-5;69D4-6	664	107	20	918	27.67%	0.26%	1.85%
Df(3L)BSC12	69F6-70A1;70A1-2	584	86	19	794	26.45%	3.46%	0.63%
Df(3L)fz-GF3b	70C1-2;70D4-5	75	10	5	105	28.57%	0.51%	2.75%
Df(3L)fz-M21	70D2-3;71E4-5	1368	154	38	1752	21.92%	-0.84%	-3.90%
Df(3L)Cat	75B4-7;75E2	354	29	19	450	21.33%	-3.20%	-4.49%
Df(3L)fz2	75F10-11;76A1-5	537	41	15	649	17.26%	0.66%	-8.56%
Df(3L)ED4782	75F2;76A1	911	101	14	1141	20.16%	-4.38%	-5.66%
Df(3L)BSC20	76A7-B1;76B4-5	146	47	5	250	41.60%	6.40%	15.78%
Df(3L)ri-Xt1	77E2-4;78A2-4	801	109	18	1055	24.08%*	4.57%	-1.74%
Df(3L)HD1	79D3-E1;79F3-6	1209	130	41	1551	22.05%	-1.31%	-3.77%
Df(3L)BSC21	79E5-F1;80A2-3	171	25	5	231	25.97%	-2.09%	0.15%
Df(3R)ME15	81F3-6;82F5-7	459	83	21	667	31.18%	3.12%	5.36%
Df(3R)3-4	82F3-4;82F10-11	570	114	25	848	32.78%	4.72%	6.96%
Df(3R)e1025-14	82F8-10;83A1-3	748	99	26	998	25.05%	0.51%	-0.77%

Df(3R)BSC47	83B7-C1;83C6-D1	520	64	19	686	24.20%	-3.21%	-1.62%
Df(3R)roe	84A6-B1;84D4-9	926	120	34	1234	24.96%	-3.90%	-0.86%
Df(3R)Cha7	90F1-4;91F5	1605	133	30	1931	16.88%	-2.62%	-8.94%
Df(3R)BSC43	92F7-93A1;93B3-6	549	77	11	725	24.28%	4.77%	-1.54%
Df(3R)e-N19	93B;94	929	174	40	1357	31.54%	0.26%	5.72%
Df(3R)ED6093	94A2;94C4	864	101	33	1132	23.67%	-5.19%	-2.15%
Df(3R)Exel6192	94B11;94D3	326	24	21	416	21.63%	-5.78%	-4.19%
Df(3R)crb-F89-4	95D7-11;95F15	1241	149	28	1595	22.19%	-2.34%	-3.63%
Df(3R)crb87-5	95F6-8;96A18-20	1232	113	34	1526	19.27%	-0.24%	-6.55%
Df(3R)Espl3	96F1;97B1	356	105	5	576	38.19%	2.99%	12.37%
Df(3R)Tl-P	97A;98A1-2	260	52	4	372	30.11%	-1.17%	4.29%

*The percentage of X NDJ is significantly higher/lower than in *X/X ; bubR1*^{D1326N}*/bubR1*^{rev1} females (multinomial-Poisson hierarchy model, P < 0.05).
[(a)] Difference between X NDJ of the deficiency-bearing flies *vs.* matched control.
[(b)] Difference between X NDJ of the deficiency-bearing flies *vs.* average controls.
