# Supplementary material for: polo Is Identified as a Suppressor of bubR1 Nondisjunction in a Deficiency Screen of the Third Chromosome in Drosophila melanogaster
Source: G3 (Bethesda). 2011 Jul 1;1(2):161–9. doi: 10.1534/g3.111.000265 (PMC3276128; doi:10.1534/g3.111.000265)
Supplement: Supporting Information [file supp_1.2.161_TableS2.pdf]

Table S2 Complementation tests of the deficiencies that affect *bubR1* X NDJ

Deficiency name used in the screen	Cytogenetic breakpoints	Cross to	Deficiency used for the complementation test	Cytogenetic breakpoints	Result of the test
Df(3L)BSC23	62E8;63B5-6	x	*Df(3L)Exel6091*	62E8;62F5	lethal
		x	*Df(3L)ED4288*	63A6;63B7	lethal
Df(3L)pbl-X1	65F6;66B7-8	x	*Df(3L)ZP1*	66A17-20;66C1-5	lethal
Df(3L)ZP1	66A17-20;66C1-5	x	*Df(3L)66C-G28*	66B8-9;66C9-10	lethal
Df(3L)BSC13	66B12-C1;66D2-4	x	*Df(3L)ZP1*	66A17-20;66C1-5	lethal
Df(3L)BSC10	69D4-5;69F5-7	x	*Df(3L)eyg[C1]*	69A4-5;69D4-6	lethal
		x	*Df(3L)iro-2*	69B1-5;69D1-6	lethal
		x	*Df(3L)ED4486*	69C4;69F6	lethal
		x	*Df(3L)Exel6117*	69D1;69E2	lethal
		x	*Df(3L)E44*	69D2;69E3-5	lethal
		x	*Syx13[01470]*	69F6	viable
		x	*Df(3L)BSC12*	69F6-70A1;70A1-2	viable
Df(3L)ED4674	73B5;73E5	x	*Df(3L)BSC561*	73A2;73C1	lethal
		x	*Df(3L)Exel6130*	73B5;73D1	lethal
Df(3L)BSC8	74D3-75A1;75B2-5	x	*Df(3L)BSC415*	74A5;75A4	lethal
		x	*Df(3L)BSC775*	75A2;75E4	lethal
Df(3L)ED4858	76D3;77C1	x	*Df(3L)Exel6136*	77B2;77C6	lethal
Df(3L)rdgC-co2	77A1;77D1	x	*Df(3L)ED4858*	76D3;77C1	lethal
		x	*Df(3L)ri-79c*	77B-C;77F-78A	lethal
		x	*Df(3L)Exel6136*	77B2;77C6	lethal
Df(3L)Pc-2q	78C5-6;78E3-79A1	x	*Df(3L)ME107*	77F3;78C8-9	viable
		x	*Df(3L)Exel9065*	78D4;78D5	lethal
		x	*Df(3L)Exel9066*	78D5;78D6	lethal
		x	*Df(3L)ED4978*	78D5;79A2	lethal
Df(3L)ED4978	78D5;79A2	x	*Df(3L)Pc-2q*	78C5-6;78E3-79A1	lethal
		x	*Df(3L)Exel9065*	78D4;78D5	viable
		x	*Df(3L)Exel9066*	78D5;78D6	lethal
Df(3L)Ten-m-AL29	79C1-3;79E3-8	x	*Df(3L)ED230*	79C2;80A4	lethal
		x	*Df(3L)HD1*	79D3-E01;79F3-6	lethal
		x	*Df(3L)Exel6138*	79D3;79E3	lethal
		x	*Df(3L)BSC21*	79E5-F1;80A2-3	lethal
Df(3R)ED5177	83B4;83B6	x	*Df(3R)Exel6144*	83A6;83B6	lethal
		x	*Df(3R)BSC549*	83A6;83B6	lethal
Df(3R)Tpl10	83C1-2;84B1-2, 83D4-5;84A4-5;98F1-2	x	*Df(3R)BSC47*	83B7-C1;83C6-D1	lethal
		x	*Df(3R)ED5197*	83B7;83D2	viable
		x	*Df(3R)Exel7284*	83C4;83D2	viable
		x	*Df(3R)Tpl6*	83D1-2;84A4-5	lethal
		x	*Df(3R)WIN11*	83E1-2;84A5	lethal
		x	*Df(3R)Scr*	84A1-2;84B1-2	lethal
		x	*Df(3R)Antp17*	84A5;84D9	lethal
Df(3R)GB104	85D12;85E10	x	*Df(3R)BSC476*	85D16;85D24	lethal
		x	*Df(3R)Exel6153*	85D19;85E1	lethal
Df(3R)ED5559	86E11;87B11	x	*Df(3R)ED5577*	86F9;87B13	lethal

		x	*Df(3R)Exel7313*	87A9;87B5	lethal
Df(3R)sbd105	88F9-89A1;89B9-10	x	*Df(3R)Exel7327*	89A8;89B1	viable
Df(3R)ED5780	89E11;90C1	x	*Df(3R)Exel6176*	89E11;89F1	lethal
		x	*Df(3R)BSC790*	90B6;90E2	lethal
Df(3R)ED5942	91F12;92B3	x	*Df(3R)BSC475*	91F12;92B4	lethal
Df(3R)BSC56	94E1-2;94F1-2	x	*Df(3R)BSC55*	94D2-10;94E1-6	lethal
		x	*Df(3R)Exel6193*	94D3;94E4	lethal
		x	*Df(3R)ED6103*	94D3;94E9	lethal
		x	*Df(3R)Exel6274*	94E4;94E11	lethal
		x	*Df(3R)Exel6194*	94F1;95A4	viable
Df(3R)mbc-R1	95A5-7;95D6-11	x	*Df(3R)Exel6194*	94F1;95A4	lethal
		x	*Df(3R)mbc-30*	95A5-7;95C10-11	lethal
		x	*Df(3R)Exel9014*	95B1;95D1	lethal
		x	*Df(3R)Exel6196*	95C12;95D8	lethal
		x	*Df(3R)crb-F89-4*	95D7-D11;95F15	lethal
		x	*Df(3R)Exel6197*	95D8;95E5	lethal
Df(3R)Exel6202	96D1;96D1	x	*Df(3R)BSC849*	96D1;96E2	lethal
		x	*Df(3R)BSC522*	96D1;96E3	lethal
Df(3R)D605	97E3;98A5	x	*Df(3R)ED6255*	97D2;97F1	lethal
		x	*Df(3R)Exel6206*	97E1;97E5	lethal
		x	*Df(3R)ED6265*	97E2;98A7	lethal
		x	*Df(3R)ED6237*	97E4;97E11	lethal
		x	*Df(3R)IR16*	97F1-2;98A	lethal
Df(3R)BSC42	98B1-2;98B3-5	x	*Df(3R)BSC498*	98A4;98B5	lethal
		x	*Df(3R)BSC499*	98A14;98B5	lethal
Df(3R)3450	98E3;99A6-8	x	*Df(3R)Exel6210*	98E1;98F5	lethal
		x	*Df(3R)ED6310*	98F12;99B2	lethal
		x	*Df(3R)ED6316*	99A5;99C1	lethal

lethal – the two deficiencies do not complement each other
viable – the two deficiencies complement each other
