# Supplementary material for: polo Is Identified as a Suppressor of bubR1 Nondisjunction in a Deficiency Screen of the Third Chromosome in Drosophila melanogaster
Source: G3 (Bethesda). 2011 Jul 1;1(2):161–9. doi: 10.1534/g3.111.000265 (PMC3276128; doi:10.1534/g3.111.000265)
Supplement: Supporting Information [file supp_1.2.161_TableS3.pdf]

Table S3 Refining the mapping of *bubR1* modifiers

Female genotype	Breakpoint	Normal progeny	Exceptional progeny		Total adjusted progeny	X NDJ
		X/XY & X/O	XX/O	O/XY		
		Df(3R)D605 –enhancer				
Df(3R)D605/+		2033	1	0	2035	0.10%
$bubR1^{D1326N}/bubR1^{rev1}$		3271	194	156	3971	17.63%
$bubR1^{D1326N}/bubR1^{rev1}$; *Df(3R)D605/+*	97E2;98A3-4	1010	213	26	1488	32.12%*
$bubR1^{D1326N}/bubR1^{rev1}$; *Df(3R)ED6255/+*	97D2;97F1	2720	157	61	3156	13.81%
$bubR1^{D1326N}/bubR1^{rev1}$; *Df(3R)Exel6206/+*	97E1;97E5	1233	107	26	1499	17.75%
$bubR1^{D1326N}/bubR1^{rev1}$; *Df(3R)ED6265/+*	97E2;98A7	990	216	118	1658	40.29%*
$bubR1^{D1326N}/bubR1^{rev1}$; *Df(3R)ED6237/+*	97E4;97E11	2648	214	98	3272	19.07%
$bubR1^{D1326N}/bubR1^{rev1}$; *Df(3R)IR16/+*	97F1-2;98A	1934	222	80	2538	23.80%
		Df(3R)ED5559 – suppressor				
$bubR1^{D1326N}/bubR1^{rev1}$		1452	149	48	1846	21.34%
$bubR1^{D1326N}/bubR1^{rev1}$; *Df(3R)ED5559/+*	86E11;87B11	270	0	0	270	0.00%*
$bubR1^{D1326N}/bubR1^{rev1}$; *Df(3R)ED5516/+*	86D8;86E13	651	61	19	811	19.73%
$bubR1^{D1326N}/bubR1^{rev1}$; *Df(3R)Exel8154/+*	86E13;86E18	582	52	16	718	18.94%
$bubR1^{D1326N}/bubR1^{rev1}$; *Df(3R)Exel7310/+*	86E18;87A1	863	85	21	1075	19.72%
$bubR1^{D1326N}/bubR1^{rev1}$; *Df(3R)ED5577/+*	86F9;87B13	1506	0	1	1508	0.13%*
$bubR1^{D1326N}/bubR1^{rev1}$; $aur^{1}/+$		1562	160	28	1938	19.40%
$bubR1^{D1326N}/bubR1^{rev1}$; $aur^{87Ac-3}/+$		1302	119	28	1596	18.42%

*The percentage of X NDJ is significantly higher/lower than in $X/X;bubR1^{D1326N}/bubR1^{rev1}$ females (multinomial-Poisson hierarchy model, P < 0.05).
